# Four Key Questions to Guide Human Rights–based Social Listening during Infodemics

**DOI:** 10.1007/s41649-024-00324-2

**Published:** 2024-12-26

**Authors:** Lisa Forman

**Affiliations:** 1 Human Rights and Global Health Equity, Dalla Lana School of Public Health, University of Toronto, Toronto, Canada; 2 Faculty of Law, University of Toronto, Toronto, Canada

**Keywords:** Infodemics, Social listening, Human rights, COVID-19

## Abstract

This paper considers what a human rights–based approach to the use of social listening to counter infodemics during a serious health threat might entail, using COVID-19 as a primary example. The paper considers social listening in the context of human rights including health, life, free speech, and privacy, and outlines what a rights-compliant form of social listening to infodemics might entail. The paper argues that human rights offer guardrails against illicit and unethical forms of social listening as well as signposts towards a more equitable, ethical, and effective public health tool. The paper first expands on the human rights dimensions of COVID-19, infodemics, and social listening. Second, it considers the human rights dimensions of social listening in relation to rights to health, life, and free speech, given international human rights law principles for limiting these rights. Finally, using this framework, the paper poses four key questions to frame a rights-based approach to social listening: Why do we listen? How do we listen? Who do we listen to and who is doing the listening? And what are the outcomes of such listening?

## Introduction

Social listening is a specific means of gathering public discourse from a variety of sources to assess and address public attitudes and perceptions on key public health issues. It is authoritatively defined as the “regular and systematic aggregation, filtering and monitoring of conversations and public discourse in a combination of traditional media, digital media, off-line and on-line sources of information that represent different populations and geographies” (Chaney et al. 2021, 5). Because of its ability to access these alternative sources, social listening offers public health practitioners a speedy means for information gathering on public health that bypasses time-consuming and cumbersome traditional methods (Picone et al. 2020). The non-traditional data collection tools used in social listening “range from sophisticated artificial intelligence and machine learning platforms to telephone hot lines, broadcast radio talk shows and documentation of community dialogues” (Chaney et al. 2021 6). Data collected through social listening can help anticipate trends in health and develop appropriate and timely public health responses. The rapidity of social listening is thus particularly well-suited to the urgency of developing public health programmes during a severe and rapid global health crisis like the COVID-19 pandemic (Picone et al. 2020; Hou et al. 2021; Chaney et al. 2021). Moreover, social listening offers an informational tool that is particularly well-suited to addressing the “infodemics” that frequently accompany epidemics or other acute health crises in the form of overabundant “information, accurate or not, in the digital and physical space” (Chaney et al. 2021, 5; World Health Organization 2023).

Social listening to various sources of health information that illustrate “people’s perceptions, attitudes, and health decisions” can enable public health practitioners to identify and close gaps “between health guidance and the population’s behaviour” (World Health Organization 2023, 1). In this light, social listening offers a critical policy tool for states to understand the nature of such infodemics and mitigate their negative impacts on public health responses to serious health threats (World Health Organization 2023), and ergo improve the efficacy of pandemic responses. In some ways, it can be considered a logical extension of standard public health surveillance methods that systematically collect, store, use, and disseminate personal information to identify an outbreak and mitigate the spread of disease (Gostin 2010). And in the same way that there have traditionally rights-based concerns about how the data collected through public health surveillance tools is used (Sekalala et al. 2020a, b), there are comparable human rights concerns about social listening.

In considering these concerns, it must be acknowledged that not responding to infodemics creates its own public health and human rights risks. The negative health impacts of the informational overloads, gaps, and confusion inherent to infodemics are clearly outlined by the World Health Organization (2023, 1):

An infodemic can promote stigma, erode trust in health authorities, affect mental health and negatively influence health decisions and behaviours, thereby making it more difficult for health authorities to respond effectively and protect the population’s health

As some of these health impacts may imply, the process and outcomes of social listening hold the capacity to realize a range of human rights of those affected, including rights to life, health, equality, participation, privacy, and free expression. These human rights place legal duties on states to first, appropriately respond to pandemics and second, they offer principles and standards to guide states in balancing urgent public health imperatives with individual rights during such an emergency, including through ensuring that “the public has accurate information about serious health threats” (Orentlicher 2021).

Yet inasmuch as social listening offers the potential to realize human rights, its misuse equally holds the capacity to violate these same rights: if the process and outcomes of social listening are not necessary to serve legitimate public health goals, if they impose disproportionate restrictions, if their use is not time-bound, or if they are used in a discriminatory way. These are the criteria proposed within international human rights law to guide justifiable restrictions of human rights, namely that such restrictions are necessary, proportionate, time-bound, and non-discriminatory (United Nations 1984). These criteria recognize that state measures (including those in service of public health) that exceed these criteria pose the risk of disproportionately harming marginalized populations and deepening power inequities. For example, social listening is misused if its purpose is to harass, persecute, silence, or stigmatize minority populations or political opposition rather than advance public health. Social listening runs afoul of human rights standards if the data collected is not anonymized or is collected without the knowledge or explicit consent of those being listened to. And social listening is not compliant with human rights if it is implemented in discriminatory ways, for example, by targeting marginalized groups in ways or for purposes unjustified by public health.

In this paper, I explore what a rights-compliant form of social listening during an infodemic might entail. I argue that human rights offer guardrails to protect against illicit and unethical abuses of social listening during an infodemic like those outlined above. I argue further that beyond guardrails, human rights offer “signposts” for equity in the form of norms, standards, and rules that can guide the formulation, implementation, and uses of social listening to protect human rights and potentially improve public health outcomes. I argue that if implemented in a rights-compliant way, social listening holds the potential to be deeply synergistic with the emphasis in human rights on participation, accountability, and non-discrimination (Potts 2008a, b), by respecting and/or including people affected by policies in their design and revision, and by building social trust in public health measures.

Yet recognizing and implementing human rights criteria in social listening face multiple broader challenges in domestic and global health governance: from assuring that non-state actors like private companies take account of human rights, to the frequently limited ability and/or willingness of states to regulate such entities, to the ability and indeed freedom of civil society and marginalized populations to challenge human rights violations and injustices by a range of state and non-state actors in this context, to the erosion of human rights in an era of growing right-wing nationalism. Recognizing these broader challenges, this paper delves into the potential contribution of human rights to assuring more ethical, equitable, and fair approaches to social listening to counter infodemics. To do so, I first expand on the human rights dimensions of COVID-19, infodemics, and social listening. Second, I focus on how social listening impacts on human rights to health, life, and free speech. I then turn to consider the criteria in international human rights law discussed above that guide state limitations of these rights. Finally, using this framework, I pose four key questions that could guide states towards a rights-based approach to social listening during a pandemic: Why do we listen? How do we listen? Who do we listen to and who is doing the listening? And what are the outcomes of such listening?

## Human Rights, COVID-19, and Social Listening

The references in this paper to human rights primarily invoke the legal concepts, standards, and rights codified and interpreted in international human rights law. In doing so, I acknowledge that this body of law is by no means the sole legal source of human rights law. I also acknowledge that law is not the sole disciplinary source of ideas and interpretations of human rights which are extensively explored in politics, philosophy, anthropology, and sociology (Da Silva 2021). While this broader context is recognized, this paper focuses specifically on the frequently binding legal rights and standards reflected in international human rights law which codifies a range of interdependent and interrelated human rights. Indeed, the modern system of international human rights law has developed so rapidly over the past decades that it is considered the fastest growing field in international law (Mutua 2008). International human rights law contains multiple human rights treaties, United Nations (UN) institutions, and human rights declarations, resolutions, conferences, and programmes of action. There are also regional human rights law systems in Africa, the Americas, and Europe, and regional human rights declarations in Asia and the Middle East.

The human rights protected in these legal systems are intended to build on and protect the central precepts of human rights law focused on protecting the inherent dignity and the equal and inalienable rights that all people have simply by virtue of being human. These human rights include civil and political rights like life, free speech, and to vote, and economic, social, and cultural rights like health, education, food, water, sanitation, and a clean and healthy environment. Moreover, virtually every state in the world is bound by at least five and often as many as 10 international human rights treaties (United Nations 2024).

International human rights law holds as a core foundational principle that all human rights are interlinked and interdependent (World Conference on Human Rights 1993). This concept recognizes that if one right is violated, those same actions are likely to violate many other human rights, and conversely, that the protection of any one human right is likely to extend protection to many other rights. This relationship is even more apparent when it comes to health and human rights which Jonathan Mann argued are intricately and inextricably interconnected and interdependent (1999). This interdependence was particularly evident during COVID-19 where state responses had extensive and interconnected human rights impacts that frequently veered into violations (Sekalala et al. 2020a, b). These linkages during COVID-19 have been described by Michael Goodhart as a form of “negative interdependence” in which a crisis triggered a “chain reaction of rights violations and deprivations that disproportionately affect oppressed and vulnerable people” (Goodhart 2020). The implication of this interdependence is that as Mann theorizes, that good public health offers a “positive interdependence” which could catalyze a chain reaction of salubrious health and human rights outcomes.

A 2020 global monitor of COVID-19 measures found that by November 2020, more than 61 per cent of all the countries in the world had implemented COVID-19 measures that were concerning from a democratic and human rights perspective because they were “either disproportionate, illegal, indefinite or unnecessary in relation to the health threat” (International Institute for Democracy and Electoral Assistance 2020). The pandemic raised questions about the impact of public health measures on human rights that human rights and public health scholars and policymakers will be analyzing for decades to come. These impacts extended from widescale restrictions of free movement inside and outside borders with lockdowns and travel restrictions, to failures to protect vulnerable populations most at risk of infection and death from COVID-19—from the elderly in long-term care homes, to the immune-compromised, people with disabilities, and the amplified risks of infection in racialized and indigenous communities, to people’s right to participate in developing and revising policy decisions that significantly impact their rights (Forman et al. 2022; Lebret 2020; Montel et al. 2020; Clay et al. 2022).

Early on in COVID-19, it became clear that policymakers needed guidance and clarity on how and where to think about human rights when it came to pandemic responses (Human Rights Watch 2020b; Puras et al. 2020). This imperative extended especially to how to think about restrictions of human rights in service of a public health emergency, a topic which has deeply engaged public discourse around the world, and which became a flash point in the ideological culture wars around COVID-19. These ideological and frequently deeply political wars saw the emergence of infodemics in which everything about COVID-19 was disputed, from its threat and severity to the efficacy of almost all public health responses to the disease, from lockdowns to masks and vaccines. While these infodemics had roots in political contestation, they were equally facilitated by big tech companies like Facebook, Google, and Twitter, whose advertising models and algorithms not only failed to interrupt but actively advanced these infodemics (Maréchal et al. 2020). For those who did not believe that COVID-19 was real, any restrictive or mandatory measures to control it did not seem either necessary or proportionate to the public health threat posed. Indeed, in North America and many parts of Europe, the tenor of much discourse on COVID-19 mandates or measures was that limiting freedom in any way was a violation of human rights (Horowitz 2022; Horowitz et al. 2021; BBC 2021).

**Fig. 1:**
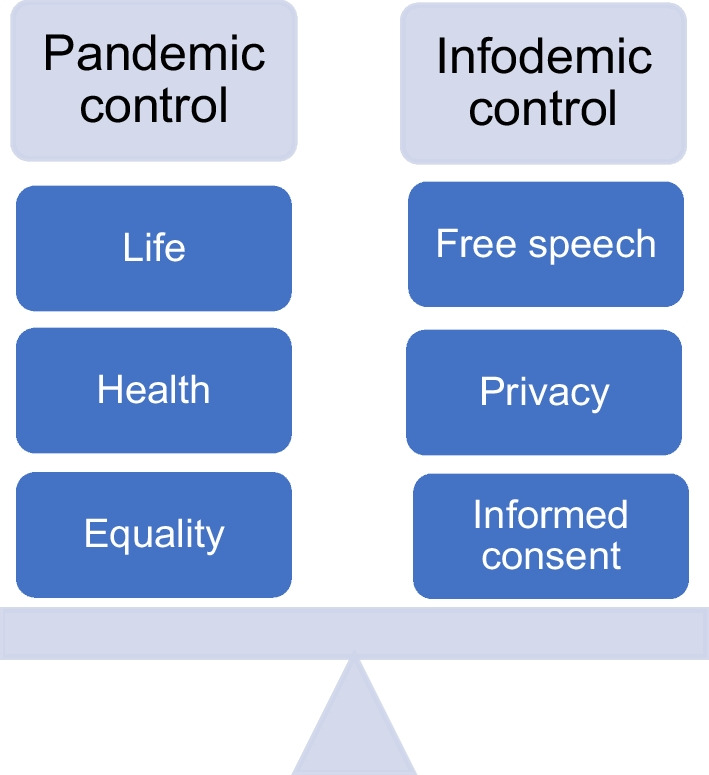
Balancing the human rights implicated in controlling pandemics and infodemics

As COVID-19 illustrates, infodemics pose major dangers to the efficacy of public health responses to pandemic disease threats and to the ability of government responses to protect both public health and people’s human rights to life and health. Conversely, a policy response to an infodemic may necessitate certain restrictions of human rights, including by pushing against the bounds of what constitutes legitimate free speech. Infodemics thus offer a classic example of the need to achieve an appropriate balance between rights in service of a legitimate public health objective and to ensure that the public has accurate information about serious health threats (Orentlicher 2021). In this respect, social listening offers a potentially powerful tool to counter the harmful impacts of infodemics and to advance diverse human rights including to health, life, and participation. The challenge for policymakers is to find the appropriate balance between these diverse rights (see Fig. 1).

Social listening allows policymakers to gather talk on social and traditional media to assess and address public attitudes and perceptions on key public health issues. To the extent that doing so supports good public health policy, social listening may be synergistic with core principles within human rights. For example, human rights law’s central focus on human dignity and equal worth has elevated three principles as holding overarching importance and cross-cutting significance, namely, participation, accountability, and non-discrimination (Potts 2008a, b). Participation is the idea that people should be able to take part in developing and revising the policies and programmes that affect them. Equally, governments must actively give voice to the people they govern and listen to what they ask for and need. Participation and the related key right of access to information are a central focus of human rights and especially of the right to health (Potts 2008b).

## Human Rights Implicated in Responding to a Pandemic

The source of state duties to address pandemic threats can be found primarily in the human rights to health and life with participation and access to information prioritized aspects of the former right. This focus has been apparent from the earliest United Nations (UN) documents on human rights and health. For example, the 1946 Constitution of the World Health Organization recognized that the enjoyment of the highest attainable standard of health is one of the fundamental rights of every human being without distinction of race, religion, political belief, economic or social condition; that governments have a responsibility for the health of their peoples which can be fulfilled only by the provision of adequate health and social measures; and that informed opinion and active co-operation on the part of the public are of the utmost importance in the improvement of the health of the people (World Health Organization 1946).

The 1948 *Universal Declaration of Human Rights* (UDHR) which established the international human rights law system outlines the key human rights implicated in responding to a pandemic. Article 3 holds that everyone has the right to life, liberty, and security of person, and Article 25 holds that everyone has the right to a standard of living adequate for the health and well-being of himself and of his family, including medical care (United Nations 1948). A right to privacy is protected in Article 12 which states that no one shall be subjected to arbitrary interference with privacy, family, home, or correspondence, nor to attacks upon honour and reputation. A right to bodily autonomy is intimated in Article 5 which prohibits torture and cruel, inhuman, and degrading treatment. A right to free speech is protected in Article 19 which states that everyone has the right to freedom of opinion and expression, which not only includes freedom to hold opinions without interference and to seek, but also, the right to receive and impart information and ideas through any media and regardless of frontiers. This latter language was drafted in 1948 in ways that appear prescient of the changes to come.

The right to life in the *Universal Declaration of Human Rights* (UDHR) is codified in the 1976 *International Covenant on Civil and Political Rights* (ICCPR) which holds in Article 6.1 that “every human being has the inherent right to life” (United Nations 1976a). This has been interpreted by the United Nations Human Rights Committee (HRC) to require states to take appropriate measures to address the general conditions in society that may give rise to direct threats to life including life-threatening diseases (United Nations 2019). The right to free expression is codified in ICCPR Article 19.2 which states that “everyone has right to freedom of expression [which] includes freedom [not just] to seek” but also to receive and impart information and ideas of all kinds. This right has been interpreted to impose a duty on state actors not to make or encourage disinformation or propaganda (United Nations General Assembly 2020). A prohibition on non-consensual medical or scientific experimentation is recognized in ICCPR Article 7, a right that echoed comparable norms then emerging in bioethics instruments like the *Nuremberg Code* and *Helsinki Declaration* in response to Nazi atrocities perpetrated in the name of medical research (Constantin 2018). This right is intimately linked to the ethical principle of “informed consent”, where “consent is considered to be free and informed when it is given on the basis of objective information from the researcher and includes not only the nature of the research, but also its potential consequences and risks involved, as well as its alternative” (Constantin 2018).

The primary source for the right to health is found in the 1976 *International Covenant on Economic, Social, and Cultural Rights* (ICESCR) in Article 12 where states recognize everyone’s right to the enjoyment of the highest attainable standard of physical and mental health (United Nations 1976b). They also undertake to take steps “to achieve the full realization of this right including those necessary for … (c) the prevention, treatment and control of epidemic, endemic, occupational and other diseases, and (d) the creation of conditions which would assure to all medical service and medical attention in the event of sickness” (United Nations 1976b).

This right has been interpreted by the UN Committee on Economic, Social and Cultural Rights (CESCR) to extend to the provision of timely and appropriate health care and to the underlying determinants of health which includes access to health-related education and information (United Nations 2000). The right to health is also interpreted as extending to the participation of the population in health-related decision-making at the community, national, and international levels (United Nations 2000). This emphasis on information and participation is threaded throughout the right to health and the duties it places on states. For example, the essential elements of the right to health are defined to include available, accessible, acceptable, and good quality health care facilities, goods, and services, and accessibility is specifically defined to include not just physical and economic accessibility but also informational accessibility without discrimination (United Nations 2000). Informational accessibility is defined as the right to seek, receive, and impart information and ideas concerning health issues and which should not impair the right to have personal health data treated with confidentiality (United Nations 2000). The right to health also extends to and is intimately related to the right to be free from “non-consensual medical treatment and experimentation” (United Nations 2000; Constantin 2018).

In ways that are relevant to both social listening and infodemics, information and participation are emphasized throughout the right to health, with states holding prioritized obligations to provide education and access to information concerning the main health problems in the community, including methods of preventing and controlling them, and to ensure that third parties like private companies do not limit people’s access to health-related information and services (United Nations 2000). The CESCR sees states as holding a range of duties relevant in this area: including to respect the right to health (don’t obstruct) which includes refraining from censoring, withholding, or intentionally misrepresenting health-related information; protecting the right to health (prevent others from obstructing) which includes ensuring that third parties do not limit people’s access to health-related information and services; and fulfilling the right to health which includes adopting measures against any threat demonstrated by epidemiological data. In other words, binding human rights law requires states to balance their duties to address a pandemic with people’s right to receive information and freely express their ideas. To the extent that achieving this balance may entail limits on other rights, these limits must be strictly curtailed.

## Human Rights Criteria for Legitimate Limitations of Rights

In international law, outside of a small handful of rights which are absolute (like the right to life and prohibitions on cruel, inhuman degrading treatment and on slavery), most rights can have reasonable limits placed on them when there are compelling public interests at stake including public health, national security, and public emergencies. These restrictions apply to a range of rights including free speech, free movement, free association, and peaceful assembly. For example, the limitations clause in the right to free speech in the ICCPR holds that:

The exercise of [the right to free expression] carries with it special duties and responsibilities. It may therefore be subject to certain restrictions, but these shall only be such as are provided by law and are necessary: (a) For respect of the rights or reputations of others; (b) For the protection of national security or of public order (ordre public), or of public health or morals. (United Nations 1976a, article 19.3)

Yet these limitations provisions are very slippery parts of human rights law, as states can use such provisions to extensively limit rights by claiming that doing so was required to protect national security or public health. This tendency frequently occurred after 9/11 where governments increasingly used security and terrorism rationales to crack down on unrelated activities including civil society activism, and to restrict ostensibly non-derogable rights like torture (Roth 2021). We have seen similar trends during COVID-19 where authoritarian governments used the pandemic as a pretext to lift democratic restrictions on their power or to crack down on civil society (Quinn 2020, Human Rights Watch 2020a). This longstanding threat prompted a group of international law experts in 1984 to develop a set of principles to guide states on reasonable and legitimate restrictions of rights (United Nations 1984). The *Siracusa Principles on the Limitation and Derogation Provisions in the ICCPR* (1984) hold that if governments are going to limit rights, such restrictions must be strictly necessary to promote general welfare in a democratic society, in that they are based on recognized grounds, respond to pressing public/social need, and pursue legitimate aims. In addition, they must be temporary, be imposed by law and not be arbitrary, they must not be discriminatory, and they must be proportional, i.e. the least restrictive alternative must be adopted where several types of limitations are available. Public health can only be used as a ground for limitations in the case of a serious threat to individual or population health, and any such measures must be specifically aimed at preventing disease or injury or providing care for the sick and injured, and give due regard to the International Health Regulations of the World Health Organization (United Nations 1984).

Thus, to use COVID-19 as an excuse to lift all rights indefinitely is a clear example of a gross violation of these precepts and an illegitimate restriction of rights. To use social listening to monitor people without their consent in order to combat COVID-19 would fall short of necessity. To use social listening as a form of public health surveillance of minorities like migrants or lesbian, gay, bisexual, transgender, and intersex (LGBTI) populations would be discriminatory if done without their consent, done without valid public health purposes, or resulting in harmful outcomes. Moreover, if data gathered through social listening is used for political purposes other than public health or to crack down on civil society, this use would fall outside the legal bounds of necessity and proportionality. As these examples indicate, these criteria offer policy-makers guidance for more equitable, ethical, and just public health policy.

## Questions to Guide Human Rights–based Social Listening

In line with the interpretations outlined above, rights-based social listening would seek to comply with the prescriptions of these diverse human rights and standards. Thus, to be human rights compliant, digital surveillance should be evidence-based, contribute to a comprehensive public health surveillance system, include sunset-clauses, be non-discriminatory, and ensure mechanisms for greater transparency and accountability, including those aimed at non-state actors such as private companies (Sekalala et al. 2020b).

An extensive scholarly interpretation of human rights principles and guidelines during a public health emergency has elaborated more fully on these duties (Habibi et al. 2023). This important interpretation holds that states must counter misinformation using human rights–compliant mechanisms and tools in general, while fully respecting and ensuring the right to freedom of expression (Habibi et al. 2023, para. 6.2.e). The principles indicate further that when a rights-based and evidence-informed public health measure results in a limitation to the right to freedom of expression, freedom of association, and the right to peaceful assembly, states must:

ensure that limitations are not used to harass, persecute, intimidate, or stigmatize persons from any particular sector of the population; and refrain from using such public health measures to silence disfavoured views, including those views that contest the necessity or legality of the measures themselves, or in any way impede the work of human rights defenders, health and care workers, journalists, insider informants (“whistleblowers”) or researchers. (Habibi et al. 2023, para 16.b)

The challenge for policymakers and civil society is thus to practically utilize these standards and interpretations to assure that social listening during infodemics effectively advances legitimate public health goals, is not abused to harass, persecute, silence, or stigmatize the populations being listened to, and is done in a transparent and respectful way. In the remainder of this paper and based upon the foregoing, I propose four key questions that could guide governments and private actors towards considering some of these criteria and assuring a more rights-based approach to social listening.

### Why is it Being Done? (Necessity)

The first question to ask is why social listening is being conducted. Is the purpose of such listening to protect public health or is public health merely a front for surveilling political opposition or gathering data to harass or detain minority groups? If social listening cannot pass this first question, it is unlikely that it could be considered human rights compliant. For instance, we have seen data from public health surveillance shared with security officials with governments surveilling telecommunications data under the guise of contact tracing (Sekalala et al. 2020a, b).

### How is it Being Done? (Proportionality)

The second question is to ask how social listening is being done? Is it being done in a way that respects rights to privacy, consent, free expression? Is data being anonymized? Is it being done transparently so that people consent to their data being used, know how it is being used, and have recourse if human rights are being violated? Is it being done in a non-discriminatory way? For example, in India, the contact tracing app Aarogya Setu became mandatory during COVID-19 making it impossible for people to consent to its use, and in Singapore, while some workers were encouraged to download the Tracetogether app, it was made mandatory for migrant workers (Sekalala et al. 2020b). Finally, is the surveillance time bound to the pandemic or does it become entrenched as standard practice of governments as we saw in the war against terror after 9/11?

### Who is Being Listened to and Who is Listening? (Non-discriminatory)

The third question asks who is being listened to. If it is the public on social media, how are the views of people not present on social media and hidden from view by digital gaps being accessed? Or are specific and already marginalized groups being surveilled that may be disproportionately harmed by surveillance? Is social listening being done by governments who are direct duty bearers under human rights and must protect human rights by ensuring that third parties are not free to indiscriminately and non-consensually surveil users of their products including through national laws to protect people from harmful uses of their data? In the absence of regulation, is an independent civil society being enabled to play a key role in developing and using accountability mechanisms including through gathering evidence of harms that can support advocacy and grassroots organizations (Maréchal et al. 2020)? Or is surveillance being conducted by private actors who hold some duties to respect human rights and who must assure that their digital surveillance does not exceed the bounds of legality outlined in human rights laws? In the case of private actors, some suggest that these duties would extend to requiring companies to engage in due diligence of the human rights impacts of their surveillance (Maréchal et al. 2020).

### What are the Outcomes of Social Listening? (Accountability)

Finally, what are the outcomes of social listening, does it advance public health or other legitimate policy objectives? If social listening falls short of serving a legitimate public health objective, who is held accountable and what redress do those whose rights have been limited have? Finally, are national legal or human rights mechanisms being used to examine how state and nonstate actors are using digital surveillance to assure human rights abuses are not taking place (Sekalala et al. 2020b).

## Conclusion

Human rights offer tools to assure that social listening to counter infodemics during serious health threats is in greater synergy with human rights standards and with human rights principles of participation, accountability, and non-discrimination. Yet the practices of many governments in COVID-19 illuminated that social listening is frequently a front for and means of violating human rights. The imperative for civil society and for policymakers is thus to assure that similar violations do not recur in future public health emergencies, and to guard against human rights violations during COVID-19 from becoming entrenched into standard state practice. Viewed in this light, we should see human rights not as optional add-ons to policy but as binding legal duties for policymakers during a public health emergency. Human rights thus are important companions to ethics when it comes to assuring more equitable and lawful public health approaches during a pandemic threat, and in providing a framework to assure more fair and effective approaches to social listening to counter infodemics.

## Data Availability

This statement is not applicable to this paper which relies on legal texts and secondary literature.
